# Clinical–Radiological Discordance in the Detection of Chronic Constipation in Children Newly Diagnosed with Autism Spectrum Disorder: A Retrospective Study

**DOI:** 10.3390/reports9040360

**Published:** 2026-10-17

**Authors:** Hamza A. Alsayouf, Redab Al-Ghawanmeh, Luay S. Alhawawsha, Osama Alsarhan, Hala Abdulsalam Hamada

**Affiliations:** 1 Dr Hamza Alsayouf Clinic, Amman 11181, Jordan; dr.osama91alsarhan@gmail.com (O.A.); hamhamnew1@gmail.com (H.A.H.); 2 Department of Pediatrics, Faculty of Medicine, The Hashemite University, Zarqa 13133, Jordan; redab@hu.edu.jo; 3 Royal Medical Services, Amman 11181, Jordan; loai_09@yahoo.com

**Keywords:** autism spectrum disorder, constipation, gastrointestinal disorders, abdominal radiography

## Abstract

**Background/Objectives:** Chronic constipation in patients with autism spectrum disorder (ASD) may be undetected during routine care and can exacerbate ASD symptoms such as irritability and hyperactivity. This retrospective study evaluated the prevalence of radiographic findings suggestive of chronic constipation in children newly diagnosed with ASD, and it compared these findings with those based on routine clinical history and examination alone. **Methods:** A retrospective chart review of pediatric patients diagnosed with ASD at neurology clinics in Jordan between 2022 and 2025 was conducted. Informed consent was required for an abdominal radiographic examination at screening to initiate the standard treatment protocol at participating clinics. A more stringent version of the Leech criteria, equivalent to a total Leech score of ≥10, was applied to the radiographic images to identify patients with findings suggestive of chronic constipation. Data were extracted via retrospective chart review using a standardized data collection form. **Results:** Among the 455 patients who met the study eligibility criteria, the mean ± SD age was 6.7 ± 3.3 years (median, 6 years; range, 2–18 years) and 80.2% were male. Most patients had radiographic findings suggestive of chronic constipation at baseline (81.5%, n = 371/455), whereas 5.3% (24/455) had findings suggestive of constipation based on their medical history and clinical examination alone. **Conclusions:** Radiographic findings suggestive of chronic constipation were common in children newly diagnosed with ASD and were identified substantially more frequently than constipation identified through routine history-taking and clinical examination in the same patients. Prospective studies are warranted to determine whether the use of alternative, standardized methods as adjunct diagnostic tools can improve clinical outcomes in this population.

## 1. Introduction

Autism spectrum disorder (ASD) is associated with difficulties with learning, communication, and social interaction, and patients present with repetitive and challenging behaviors [1]. Many individuals with ASD also present with gastrointestinal (GI) disorders, such as constipation, flatulence, gastroesophageal reflux, abdominal distension, irritable bowel syndrome, abdominal pain, vomiting, and diarrhea, and the severity of these disorders is known to correlate with the severity of patients’ core ASD symptoms [2,3,4,5].

Individuals with ASD are over four-fold more likely to develop GI disorders than neurotypical individuals [6,7]. Given the high comorbidity of GI disorders and their known link to symptom severity in patients with ASD, many clinicians consider it important to manage these disorders as part of an overall treatment strategy for the management of ASD [2,5,8]. However, GI symptoms in patients with ASD are challenging to diagnose, particularly in pediatric populations, and are therefore likely to be undertreated [4,9]. Particularly in nonverbal children or those with cognitive impairment, classic GI symptoms such as straining or painful defecation may be entirely absent or replaced by behavioral signs such as aggression, irritability, or self-injury, which can mislead clinicians and delay diagnosis [4]. In such cases, the sensitivity of routine clinical abdominal examination and history-taking is markedly reduced, necessitating the use of alternative diagnostic tools to identify underlying gastrointestinal issues [10,11].

Constipation is one of the most commonly reported GI disorders among patients with ASD, and the likelihood of this disorder increases with the severity of social and verbal impairment [7,12]. GI disorders such as constipation have been shown to exacerbate the symptoms of ASD, such as anxiety, irritability, hyperactivity, social withdrawal, and sleep disturbances [4]. Notably, a large proportion of patients with ASD with constipation tend to have functional constipation, i.e., constipation that is not clearly due to any overt pathology [12]. Children with ASD are more likely than neurotypical children to present to emergency departments for constipation, and they are also more likely to be admitted for this condition [13]. Supplements and therapeutic diets (e.g., ketogenic, gluten-free, or casein-free diets, or diets rich in antioxidants and/or probiotics) and behavioral treatment strategies may assist in managing GI symptoms, although further research is needed [5,14]. It is important to also monitor for GI symptoms arising as adverse effects of medications used to manage ASD [15].

Constipation in children is typically defined clinically rather than radiographically. Based on the Rome IV criteria for children aged 4 years and older, functional constipation is diagnosed when at least two of the following have been present for at least 1 month and these symptoms cannot be explained by any other medical condition: ≤2 defecations in the toilet per week; ≥1 episode of fecal incontinence per week; a history of retentive posturing or excessive volitional stool retention; a history of painful or hard bowel movements; the presence of a large fecal mass in the rectum; and a history of large-diameter stools that may obstruct the toilet [16]. Chronic constipation is distinguished from acute constipation by the persistence of these features over time. Importantly, these criteria depend on the reporting of symptoms and behaviors; this requirement is difficult to satisfy in children with limited verbal ability or atypical sensory perception.

Several screening tools have been developed to help diagnose constipation and other GI disorders in patients with ASD. These include the 35-item Autism Speaks–Autism Treatment Network GI Symptom Inventory [17]; a condensed, 17-item format of this inventory [18]; the ASD Gastrointestinal and Related Behaviors Inventory (ASD-GIRBI) [19]; and the Gastrointestinal Symptom Severity Scale (GSSS), which is a 7-item tool based on the Rome IV criteria [20]. Rather than relying on patients being able to verbalize and localize symptoms, the above questionnaires rely on observable signs that caretakers can identify. However, the sensitivity and specificity of these tools could benefit from further refinement [20].

Radiographic assessment of fecal loading has been widely used in pediatric research and continues to be employed in clinical practice, particularly when clinical assessment is limited or inconclusive [21,22,23,24]. However, the diagnostic utility of abdominal radiography for childhood constipation remains debated. A systematic review of six studies on the use of abdominal radiography to diagnose constipation in neurotypical children reported sensitivities of 60% to 80% and specificities of 43% to 99% [24], while a more recent systematic review of three studies in children published between 2012 and 2022 reported sensitivities of 73% to 92%, specificities of 26% to 92%, and overall diagnostic accuracies of 78% to 90% [25]. In both systematic reviews, the authors noted substantial heterogeneity in study design, radiographic parameters, and the clinical criteria used to define a positive diagnosis against which the diagnostic performance of radiography was assessed. In neurotypical populations, clinical assessment is a reasonable comparator; however, its applicability to children with ASD, in whom this comparator performs poorly, is uncertain [4,9,20].

Currently, abdominal X-rays are typically not performed routinely in patients upon diagnosis with ASD but are instead considered if indicated by the patient’s medical history or clinical examination, or where a clinical examination cannot be performed or is inconclusive [10,21,22,23,24]. Given the high comorbidity of chronic constipation with ASD [2,5,8,9], the limitations faced by standard diagnostic methods in this population [4,9,20], and the potential impact of constipation on patient outcomes [4], we performed a retrospective chart review for all patients diagnosed with ASD at our clinics who had consented at screening to an abdominal X-ray as part of initiating the broader treatment protocol. The objective of this study was to determine the baseline prevalence of radiographic findings suggestive of chronic constipation in children newly diagnosed with ASD, and to compare these findings with constipation identified by routine medical history-taking and clinical examination in the same patients.

## 2. Materials and Methods

### 2.1. Patients

This report describes a retrospective chart review of all pediatric patients with ASD who were diagnosed and treated at the authors’ neurology clinics in Jordan between 2022 and 2025. Patients were diagnosed with ASD per the Diagnostic and Statistical Manual of Mental Disorders (DSM)-5 criteria [26] by a pediatric neurologist, and informed consent was requested from all parents/caregivers at screening for the patients to undergo an abdominal X-ray as part of the broader consent to initiate the standard ASD treatment protocol at each clinic. In our clinical setting, abdominal radiography was performed as part of a structured baseline intake protocol at the initial visit and was not obtained solely for research purposes.

One patient with Hirschsprung disease underwent diagnosis at one of the participating clinics during the time period of this study, but they were not recommended to initiate the treatment protocol; therefore, they were not included in this study. None of the patients included in the retrospective chart review had a diagnosed organic GI disease.

### 2.2. Patient Histories and Clinical Examinations

Prior to the patient’s abdominal X-ray, their history was collected regarding bowel movements (frequency, consistency, pain, withholding behavior, diet, fluid intake, medications, and prior treatment for constipation) to evaluate their risk for constipation, and a physical exam of the abdomen was undertaken to evaluate for distension and palpable fecal masses. These signs and symptoms of constipation were reported as part of the standard ASD treatment protocol applied at the participating clinics. Perianal examinations, digital rectal examinations, and underwear inspection were not performed routinely, given that many children with ASD find this too distressful due to issues such as tactile sensitivity, difficulties in understanding the purpose of the examination, and a limited capacity to give or withhold assent.

### 2.3. Abdominal Radiography

Abdominal radiographs were interpreted by a trained pediatric radiologist who was blinded to the patients’ clinical history (one radiologist for all patients), using a modified version of the Leech criteria [21].

For the application of the Leech criteria for the diagnosis of chronic constipation in neurotypical children, the colon is divided into three sections: right colon, left colon, and rectosigmoid colon. Each section is assigned a visual score from 0 to 5 based on the degree of fecal loading and bowel dilatation (from 0 = no feces visible, to 5 = severe fecal loading with bowel dilation), with a total score of ≥9 considered indicative of chronic constipation [21]. In the present study, a more stringent definition of chronic constipation was applied, requiring fecal loading of >50% together with bowel dilation in at least two colonic sections, equivalent to a total Leech score of ≥10. As in the Leech scoring system [21], bowel dilation was defined as a visibly enlarged colonic caliber relative to expected normal anatomy, and any loss of a normal haustral pattern was also noted. Rectal fecal impaction and the absence of normal bowel gas patterns [27] were also assessed.

It should be noted that these radiographic criteria characterized fecal loading at a single time point and were not in themselves diagnostic of functional constipation. The duration of symptoms was not recorded over time, and chronicity was inferred from the clinical context of the presentation rather than established radiographically. Thus, throughout this report we describe radiographic results as findings ‘suggestive of’ chronic constipation rather than as diagnostic thereof. Where patients were suspected to have chronic constipation, they were referred to a pediatric gastroenterologist for management.

### 2.4. Data Collection and Analysis

Baseline characteristics were obtained through retrospective review of medical records, including demographic data, clinical presentation, relevant medical history, and abdominal radiography. Patients were evaluated and managed according to the standard clinical protocol at our clinics, which included initial assessment and appropriate investigations, followed by structured treatment and regular follow-up.

Given the retrospective nature of this study, data on ASD severity at baseline was not available for all patients. To evaluate ASD severity, our clinics make use of a full, in-depth neuropsychiatric assessment (NSA) based on standardized instruments, and many patients’ families did not opt for a full NSA due to cost. For these reasons, the data collected in this study could not be stratified by ASD severity.

Data for this retrospective chart review were extracted using a standardized data collection form. This study was exploratory and all data were analyzed descriptively. Confidence intervals were calculated with the VassarStats online statistical computational tool [28].

## 3. Results

A total of 455 patients were included in this retrospective chart review (2022 to 2025), comprising all patients who were diagnosed with and treated for ASD during this time period at the participating clinics. The patients’ mean ± SD age was 6.7 ± 3.3 years (median, 6 years; range, 2–18 years) and 80.2% were male (Table 1).

Radiographic findings of chronic constipation were reported for 81.5% (n = 371/455; 95% confidence intervals [CI]: 77.7% to 84.8%) of patients prior to initiating treatment for ASD (Table 1). In contrast, only 5.3% (24/455; 95% CI: 3.6% to 7.7%) of patients had or reported signs suggestive of this condition based on their routine clinical examination and medical history; all of these patients were also identified by radiography. The agreement between radiographic findings and clinical presentation was thus 100% for negative findings (n = 84/84) and 6.5% for positive findings (n = 24/371), with an overall categorical agreement of 23.7% (n = 108/455; 95% CI: 20.1 to 27.9%; Cohen’s κ: 0.025). The distribution of patients across subgroups based on age and sex and their corresponding radiographic findings are presented in Table 2; no marked differences were noted across the age and sex subgroups.

## 4. Discussion

This retrospective study among 455 patients diagnosed with ASD, prior to initiating treatment, indicated a much higher prevalence of findings suggestive of chronic constipation as evaluated by abdominal X-ray (81.5%) versus that suggested by these patients’ medical histories and clinical examinations at screening (5.3%).

This clinical–radiologic discordance suggests that relying solely on history-taking and physical examinations may underestimate the rate of constipation in children with ASD. Previous studies have similarly reported a poor correlation between parental/caregiver reports, clinical examinations, and objective diagnostic tools [9,12,20]. Radiographic evaluation may be of particular value when clinical uncertainty persists, especially in children with communication limitations or atypical symptom presentation. Although radiographic findings alone do not provide definitive evidence of constipation, they may serve as an adjunct to clinical assessment to help identify patients in whom further clinical evaluation is warranted.

A recent meta-analysis of clinical and observational studies reported the prevalence of GI disorders to be between 0% and 69% (general prevalence, 33%) among patients with ASD, with significant heterogeneity observed in the methods used in each study to assess GI function (most studies were mainly questionnaire-based) [29]. However, patients with ASD, and children in particular, may be unable to verbalize and localize GI symptoms, and they may not present with clear signs of discomfort or pain [4,8]. Instead, such patients may present with nonspecific signs such as irritability, aggression, sleep disturbances, repetitive behaviors, and hyperactivity. It is this group of patients that are at greatest risk for remaining untreated.

Beyond the data collected as part of this retrospective study, our clinical experience with this study cohort suggested that caregivers of children with ASD tend to report no constipation if their child passes stool daily or every other day, regardless of the quantity passed, the consistency of the stool, or associated symptoms such as excessive flatulence or abdominal distention. This aligns with published findings that caregiver and child reports of constipation frequently differ, and that applying the Rome IV criteria—which account for stool consistency, painful defecation, and incomplete evacuation rather than frequency alone—identifies significantly more cases of constipation than a single frequency-based question [30]. We also noted that families tend to regard a change from their child’s baseline bowel pattern as the primary indicator of constipation. That is, if a child has passed small amounts of stool every other day for years, the caregiver only considers constipation to be present when this established baseline changes. This observation likely reflects the well-documented difficulty of recognizing gastrointestinal symptoms in children with ASD, given their limited verbal ability and atypical sensory perception, which make it challenging for both child and caregiver to identify and communicate discomfort [7]. Compounding this, bladder and bowel dysfunction—despite being common in children with ASD—is frequently unrecognized in this population [31]. Consistent with these gaps, many families of children with confirmed chronic constipation in our study were surprised when informed of the diagnosis, with a common response being that the child had been passing stools daily. These families were unaware that daily stool passage does not necessarily indicate complete bowel evacuation. This is consistent with the phenomenon of functional fecal retention (FFR), which occurs frequently in autistic individuals and is characterized by regular stooling with incomplete evacuation, such that the bowel is never fully emptied; this can lead to symptoms such as abdominal pain, bloating, and toileting accidents despite an apparently normal stooling frequency [32].

Although the etiology of GI disorders in ASD is not yet fully elucidated, various factors are thought to contribute, including genetics, dysregulation of the immune system, an abnormal gut microbiome, high oxidative stress, reduced GI motility, and increased intestinal permeability [4,5,33,34,35,36]. In addition, the sensory sensitivities and behavioral challenges common among patients with ASD can exacerbate GI symptoms by leading to toilet avoidance and food selectivity [5,36]. In turn, GI disorders can exacerbate ASD symptoms by affecting behavior, thereby creating a vicious cycle [2,3,4,5,14,33,35]. In fact, it remains unclear whether having ASD makes patients more susceptible to GI disorders, or whether chronic GI disorders, particularly in early childhood, increase the risk of neurodevelopmental delay and ASD [3].

The reported radiation burden of a single abdominal radiograph in a child is approximately 0.05 mSv [37]; older surveys spanning a wider range of equipment and techniques report effective doses of between 0.03 and 0.77 mSv [38]. For context, this is comparable to several days to a few weeks of natural background radiation, and this is roughly two orders of magnitude below the dose of an abdominal CT examination. Nonetheless, the risk of cumulative radiation exposure should be carefully weighed against the potential benefits for each patient.

There are alternative modalities to radiography that were not assessed in the present study. Transabdominal ultrasound is the most relevant comparator: it involves no ionizing radiation, is well tolerated, and can be used to measure transverse rectal diameter and identify fecal impaction [39]. A recent systematic review and meta-analysis reported a pooled sensitivity of 0.68 (95% CI 0.55–0.78) and specificity of 0.81 (95% CI 0.71–0.88) across seven studies for the diagnosis of functional constipation, and a sensitivity and specificity of 68% to 100% and 83% to 100%, respectively, across five studies for the identification of fecal impaction [40]. These figures are broadly comparable to those reported for abdominal radiography, and the absence of radiation exposure is a material advantage in a population that may require repeated assessment over time. Against this, ultrasound is operator-dependent and requires the child to tolerate probe contact (which may be difficult where tactile sensitivity is present, as is common in ASD). Transabdominal ultrasonography may also be limited by technical factors, including bowel gas and inadequate bladder distension, which can impair visualization of the rectum and the reliability of rectal diameter measurements [39]. Magnetic resonance imaging is not a practical alternative in this setting, as it requires patients to remain still for a lengthy period of time.

Overall, while acknowledging concerns regarding radiation exposure, our findings suggest that the incorporation of alternative modalities such as radiography may be beneficial as an adjunct tool within the broader multidisciplinary assessment of patients with newly diagnosed ASD. Future research should explore whether the early detection and management of constipation using such a multidisciplinary approach can lead to improved behavioral outcomes and decreased healthcare utilization in individuals with ASD. Additionally, prospective studies are recommended to examine the relationship between chronic constipation treatment and quality-of-life improvement indicators in this population.

This study had several limitations, including its retrospective design and the inherent lack of a neurotypical control group. All radiographs were reviewed by a single board-certified pediatric radiologist who was blinded to the patients’ clinical history and who applied predefined criteria. While this approach ensured internally consistent image interpretation, the possibility of systematic observer bias cannot be excluded. Multi-observer scoring with formal reliability statistics is necessary to establish the reproducibility of these findings and should be built into prospective studies in this population. While a modified version of the Leech scale was applied here, scoring systems such as those by Leech and Barr [21,22] have not been validated for children with ASD; standardized scoring systems specific for this population are needed to ensure greater rigor in future studies. Currently, there is conflicting evidence regarding the association between the clinical symptoms of functional constipation and fecal loading on abdominal radiographs, and there is also no clinical consensus regarding the broader application of abdominal radiographic features to diagnosis [23,24,41,42,43,44,45]. Additionally, perianal and digital rectal exams could not be routinely performed. Thus, it is possible that the current findings overestimate the clinical–radiologic discordance reported here.

A further limitation is that the radiographs were reported using a composite score rather than as segment-level subscores. This means that the distribution of fecal loading across the right, left, and rectosigmoid colon could not be characterized, and the radiographic findings could not be stratified by the chronicity of constipation. Segment-level scoring and symptom duration should be captured prospectively in future studies, as these variables may help identify which radiographic patterns are most closely associated with clinically meaningful constipation in this population. A lack of subgroup analyses based on patients’ ASD severity and ability to communicate symptoms also limits interpretation of the proposed clinical–radiologic discordance–this would be a key analysis to perform in future studies, as this discordance may be higher for patients with greater ASD severity. The study was conducted at three clinics within the same region, and the results may not be fully generalizable to other populations and healthcare systems. Finally, the potential influence of factors such as diet, supplements, probiotics, laxatives, physical exercise, and socioeconomic circumstances was not analyzed, and the results could not be adjusted for these factors. Prospective studies using validated methods in larger populations are needed to fully evaluate the prevalence of chronic constipation in patients diagnosed with ASD.

## 5. Conclusions

Using abdominal radiography, this retrospective study identified findings suggestive of a high prevalence of chronic constipation among children newly diagnosed with ASD. This prevalence was markedly higher than that identified by routine history-taking and clinical examination in the same patients, indicating substantial clinical–radiological discordance. These findings indicate that clinicians should maintain a low threshold for further investigation in children newly diagnosed with ASD, particularly for those who are nonverbal or who present with behavioral changes that may represent unrecognized gastrointestinal discomfort.

The retrospective nature of this study precludes a recommendation for universal radiographic screening–such a recommendation would require prospective evidence that early radiographic detection improves clinical outcomes, and a formal weighing of potential benefits versus the risk of cumulative radiation exposure. What our findings do support is that relying on patient history and physical examination alone is insufficient in children with ASD, and that objective assessment–either by radiography where clinically justified, or potentially by ultrasound where available–deserves prospective evaluation as part of a multidisciplinary assessment of patients with newly diagnosed ASD.

## Figures and Tables

**Table 1:** Baseline demographic and clinical characteristics of the study population.

Characteristic	Value
Total patients, n	455
***Age, years***	
Mean ± SD, years	6.7 ± 3.3
Median, years	6
Age range, years	2–18
Male, n (%)	365/455 (80.2)
***Radiographic Findings***	
Radiographic finding suggestive of chronic constipation, n (%)	371/455 (81.5)
***Clinical Assessment***	
Signs/symptoms of constipation per medical history and clinical examination, n (%)	24/455 (5.3)
***Agreement between assessments***	
Agreement between positive radiographic findings and clinical assessments, n (%)	24/371 (6.5)
Agreement between negative radiographic findings and clinical assessments, n (%)	84/84 (100)
Overall agreement ^1^ between radiographic findings and clinical assessments, n (%)	108/455 (23.7)

^1^ Overall agreement was calculated based on patients having radiographic findings (whether suggestive of chronic constipation or not) that correlated with findings based on medical histories and clinical examinations alone. SD, standard deviation.

**Table 2:** Findings suggestive of chronic constipation in patients grouped by age and sex.

Age Group (years)	Radiographic Findings Suggestive of Chronic Constipation, n (%)	Agreement ^1^ Between Radiographic Findings and Clinical Presentation, n (%)
*Grouped by age (years)*		
2–6 (n = 268)	218 (81.3)	64 (23.9)
7–12 (n = 150)	121 (80.7)	36 (24.0)
13–18 (n = 37)	32 (86.5)	8 (21.6)
*Grouped by sex*		
Male (n = 365)	292 (80.0)	90 (24.7)
Female (n = 90)	79 (87.8)	18 (20.0)

^1^ Agreement was calculated based on patients having radiographic findings (whether suggestive of chronic constipation or not) that correlated with findings based on medical histories and clinical examinations alone.

## Data Availability

The anonymized data presented in this study are available upon reasonable request from the corresponding author.

## Abbreviations

The following abbreviations are used in this manuscript:

ASD	Autism spectrum disorder
CI	Confidence intervals
GI	Gastrointestinal
SD	Standard deviation
